# Investigation of Mechanical Properties and Color Changes of 3D-Printed Parts with Different Infill Ratios and Colors After Aging

**DOI:** 10.3390/ma17235908

**Published:** 2024-12-03

**Authors:** Oğuz Koçar, Nergizhan Anaç, Erhan Baysal, Furkan Parmaksız, İrfan Akgül

**Affiliations:** 1 Department of Mechanical Engineering, Faculty of Engineering, Zonguldak Bülent Ecevit University, Zonguldak 67100, Türkiye; oguz.kocar@yahoo.com.tr (O.K.); f.parmaksiz@beun.edu.tr (F.P.); 2 Alaplı Vocational School, Zonguldak Bülent Ecevit University, Zonguldak 67850, Türkiye; erhanbaysal@beun.edu.tr; 3 Scientific and Technological Research Center, Duzce University, Duzce 81620, Türkiye; irfanakgul@duzce.edu.tr

**Keywords:** 3D printing, PLA, aging, mechanical properties, total color difference

## Abstract

Since their inception, plastics have become indispensable materials. However, plastics used for extended periods in industrial applications are prone to aging, which negatively impacts their material behavior and performance. To ensure the long-term usability of these materials, they must be tested in real-time, in-service environments to assess degradation. In practice, however, accelerated aging techniques are commonly employed to avoid time loss. Over time, various indicators of degradation in plastics emerge, such as changes in molecular weight, cracking, and mechanical properties like strain at break and impact strength. Among these, color deterioration or change is a critical factor that helps evaluate the service life of these materials. Considering the increasing use of plastics in 3D printing today, and the growing focus on strength over aesthetics in these applications, it is particularly useful to evaluate aging in plastics based on the relationship between color and strength. The wide application of 3D printing in various industries necessitates understanding material properties under aging conditions. This study examines the effects of aging on the mechanical behavior of polylactic acid (PLA) with three different colors (yellow, orange, and red) and three different infill ratios (20%, 60%, and 100%). The samples underwent an accelerated aging process of 432 h, which included 8 h of UV radiation, 15 min of water spraying, followed by 3 h and 45 min with the UV lamps turned off. Tensile tests, bending tests, hardness measurements, and color evaluations were conducted on the samples, linking the color changes after aging with the materials’ mechanical properties. The results show that after aging, yellow samples with a 100% infill ratio exhibited a 6.9% increase in tensile strength (44.50 MPa to 47.58 MPa). Orange samples with a 100% infill ratio were less affected by aging, while red samples experienced a decrease in tensile strength across all infill ratios. Regarding bending force, increases were observed in the orange, yellow, and red samples by 10.37%, 25.05%, and 8.87%, respectively. This study underscores the importance of color selection when designing 3D-printed materials for long-term applications.

## 1. Introduction

Additive manufacturing, one of the rapidly advancing technologies in line with Industry 4.0, is expanding its application areas daily. This growth is driven by the numerous advantages that additive manufacturing technology offers over traditional production methods. These benefits include reduced time from design to the final product, rapid adaptability to design changes, ease of producing complex geometries, and cost-effectiveness. Among the various types of additive manufacturing, the most widely used method involves producing parts with 3D printers, specifically through fused deposition modeling (FDM). In the 3D printing process, parts are fabricated by melting thermoplastic-based materials, known as filaments, at specific temperatures and depositing them layer by layer. The properties of the final product depend on factors such as the type of filament material used [1,2,3], printing parameters [4,5], and the material’s color [6,7]. Since the properties of parts produced by additive manufacturing differ significantly from those created through traditional manufacturing, ensuring material safety and understanding their potential applications are critical [8]. In everyday life, products made with 3D printers are frequently utilized by both home users and industrial enterprises. These products are often exposed to environmental factors such as temperature, humidity, corrosion, and ultraviolet light, which can alter their material properties [9]. Studies investigating the effects of environmental conditions on polymers have reported that mechanical properties degrade after accelerated aging. For instance, exposing PLA and PETG materials to 24 h of dry UV-B and UV-C, and ABS-PC materials to 24 h of UV-C, led to mechanical property reductions [10,11,12]. Additionally, it has been noted that the color of PLA, PETG, ASA, and ABS materials significantly influences their mechanical properties after aging [13]. Given that additive manufacturing materials are subject to diverse operating conditions, including variable weather, it is crucial to develop reliable predictions regarding their long-term performance [14,15].

Unlike metals, degradation in plastics occurs as a gradual breakdown of molecular chains, leading to changes in texture and structure [16]. This degradation results in brittleness, discoloration, and the loss of certain physical properties [17,18,19]. Due to the high costs and time demands of long testing durations in aging studies, the industry has consistently shown interest in shorter testing processes. As a result, accelerated aging procedures (including both physical and chemical aging methods) are commonly applied to polymers to predict degradation in advance. Numerous studies in the literature address the aging of 3D-printed materials [20,21]. In one such study, Amza et al. investigated the aging of 3D-printed PLA and PETG polymers under 24-h UV-B radiation. After aging, both PLA and PETG parts exhibited reductions in tensile strength (PLA: −5.3%; PETG: −36%) and compressive strength (PLA: −6.3%; PETG: −38.3%), while hardness values remained largely unchanged. Visual inspections revealed that the aged PLA samples (blue, opaque) became shinier, whereas PETG samples (natural, transparent) developed a yellowish tint [11]. In another study focused on PLA aging, samples were exposed to UV radiation for 4, 6, and 8 weeks at 45 °C and 65% relative humidity. The results demonstrate that as UV exposure duration increased, both the Young’s modulus and compressive strength of the material also increased [22]. Similarly, in a study involving a 3D-printed ABS–PC copolymer (a blend of acrylonitrile butadiene styrene and polycarbonate), samples were subjected to 24 h aging under UV-C and UV-B light. The copolymer showed color changes consistent with the degradation of its ABS component, accompanied by a decline in mechanical properties post-aging [10]. A different study examining color changes due to aging compared uncolored ABS (natural) and dark gray ABS. Both sample types exhibited significant yellowing with extended aging time, but the uncolored material experienced the most severe color deterioration. The study also noted that surface hardness increased with prolonged aging [23]. Varsavas and Kaynak investigated the performance of PLA and PLA/GF (glass fiber-reinforced PLA) composites under UV radiation. They reported that aging caused a whitening effect in PLA, whereas the yellowish-gray color of the PLA/GF composite faded. Additionally, they observed that reductions in mechanical properties, particularly in strength and toughness, were more pronounced in pure PLA than in the PLA/GF composite [24].

Gao et al. [25] demonstrated in their tests using ABS and PLA materials in eight different colors that filament color significantly influences the mechanical properties of 3D-printed parts, including tensile strength, compressive strength, and flexural strength. While these studies primarily focused on the mechanical properties or color changes of materials after aging, the combined effects of aging on 3D-printed materials with varying infill ratios, as well as the relationship between color changes and strength, have not been thoroughly explored.

In many cases, the relationship between filament color and the quality of 3D-printed products has been overlooked [26]. However, Spencer et al. [27] noted that color enables the rapid and direct visualization of heterogeneous oxidation in polymers, suggesting that optical properties can serve as a practical method for assessing polymer degradation. Similarly, Moreno Nieto et al. [28] observed color changes in PLA resulting from hydrolysis degradation under saline conditions at 20 °C, recommending further investigation to better understand the link between color changes and mechanical property variations in 3D-printed parts.

Song et al. [29] suggested that the strength loss observed in composites after thermal aging might be related to color change. Additionally, it is well-established that colorants can affect the structural integrity and performance of polymers [30]. However, the relationship between color and plastic properties is often complex [31]. This complexity has drawn attention, prompting the authors of the present study to focus on the criteria linking color and performance in 3D-printed PLA materials with varying infill ratios and colors under aging conditions.

In this study, the effects of aging on the mechanical behavior of 3D-printed PLA materials were investigated with respect to color and infill ratio. PLA sheets in three colors (yellow, orange, and red) and three different infill ratios (20%, 60%, and 100%) were produced using a 3D printer. The samples underwent an accelerated aging process lasting 432 h, following the ISO 4892-3 standard for lifetime assessment. After aging, the hardness values, tensile strength, and bending force of the samples were measured. Additionally, color measurements were taken, and the relationship between mechanical properties and color change was analyzed. The findings from this research provide valuable insights into the strength–color relationship in 3D-printed materials, contributing to their optimization for long-term applications.

## 2. Material and Method

### 2.1. Properties of Filament

Filameon brand (Kayseri, Türkiye) PLA filaments were used in this experiment. PLA is a type of filament widely utilized in 3D printing systems due to its advantages, such as ease of production and simplicity in the 3D printing process [32]. PLA materials are derived from organic sources like corn starch and sugarcane, making them biodegradable and suitable for environmentally friendly manufacturing practices. Additionally, PLA is biocompatible, meaning it poses no harm to human health [33,34,35]. These characteristics made PLA the ideal choice for producing the experimental specimens. However, despite its many advantages, PLA has weak UV resistance due to its chemical structure. When exposed to natural or artificial UV light sources, its physical and chemical properties deteriorate, resulting in a reduction in mechanical performance [36,37]. The primary reason for selecting yellow and red colors for this study is that they are primary colors, and their combination produces orange. The mechanical properties provided by the filament manufacturer are presented in Table 1.

### 2.2. 3D Printing Processing

The 3D printing method known as fused deposition modeling (FDM) was used to produce the samples in the experiment. The 3D-printed products were manufactured using the Ender 3S-1 model device from the Creality brand (Shenzhen, China). Prior to production, the samples were designed using SolidWorks 2023 computer-aided design (CAD) software and then converted into STL format to make them compatible with 3D printing. The solid sample models were sliced using Ultimaker Cura 5.7.0 software according to the parameters provided in Table 2. The samples were printed in a horizontal orientation on the build plate without support structures. The printing parameters remained constant for all samples throughout the 3D printing process.

Due to their structure, PLA materials are prone to moisture absorption. To minimize the impact of external environmental factors, the produced samples were stored in a moisture-free environment and kept away from sunlight.

### 2.3. Aging Process

The plastic samples were subjected to an accelerated aging period of 432 h using the QUV (Accelerated Weathering Tester) device from Q-lab (Westlake, OH, USA), in accordance with the ISO 4892-3 standard [39]. The process involved 8 h of UV radiation, followed by 15 min of water spraying, and then 3 h and 45 min during which the UV lamps were turned off while the water spraying process continued. This procedure was conducted at 50 °C with UV fluorescent lamps providing 50 MJ/m^2^ of radiation. During the condensation phase, the relative humidity was maintained at (50 ± 1)% [11,40].

### 2.4. Determining the Color Scale of the Parts

The color measurements of the test samples were conducted using the CHN-SPEC CS-410 device with the CIE Lab* color system. The CIE Lab* and ∆E* were introduced by the International Commission on Illumination (CIE) in 1976 [41]. In the CIE color system, color is represented by three coordinates: “L*, a*, and b*”. According to this system, the L* (lightness–darkness), a* (green–red), and b* (blue–yellow) parameters of the samples were measured before and after aging. Each measurement was repeated three times, and the color of the samples was determined by averaging these three measurements. Reference color measurements were taken from unaged samples, while other color measurements were taken from aged samples. To express the color difference of the parts as a single value, the average values of Δa*, Δb*, ΔL*, and ∆E* were calculated using the relevant formulas in Equations (1)–(5). The total color difference, ΔE*, between two specimens is calculated as the distance between their positions in the CIELAB color space.

(1) $${\Delta E}^{*}=\sqrt{{\left({\Delta L}^{*}\right)}^{2}+{\left({\Delta a}^{*}\right)}^{2}+{\left({\Delta b}^{*}\right)}^{2}}$$

(2) $${\Delta E}^{*}=\sqrt{{\left(L_{2}^{*}-L_{1}^{*}\right)}^{2}+{\left(a_{2}^{*}-a_{1}^{*}\right)}^{2}+{\left(b_{2}^{*}-b_{1}^{*}\right)}^{2}}$$

(3) $${\Delta L}^{*}=L_{2(aged\;specimen)}^{*}-L_{1(unaged\;specimen)}^{*}$$

(4) $${\Delta a}^{*}=a_{2\;(aged\;specimen)}^{*}-a_{1\;(unaged\;specimen)}^{*}$$

(5) $${\Delta b}^{*}=b_{2(aged\;specimen)}^{*}-b_{1(unaged\;specimen)}^{*}$$

where ΔL*, Δa*, and Δb* represent the differences in the L*, a*, and b* values between the color of the unaged samples and the color of the aged samples.

### 2.5. Tests Used to Determine Mechanical Properties

**Figure 1:**
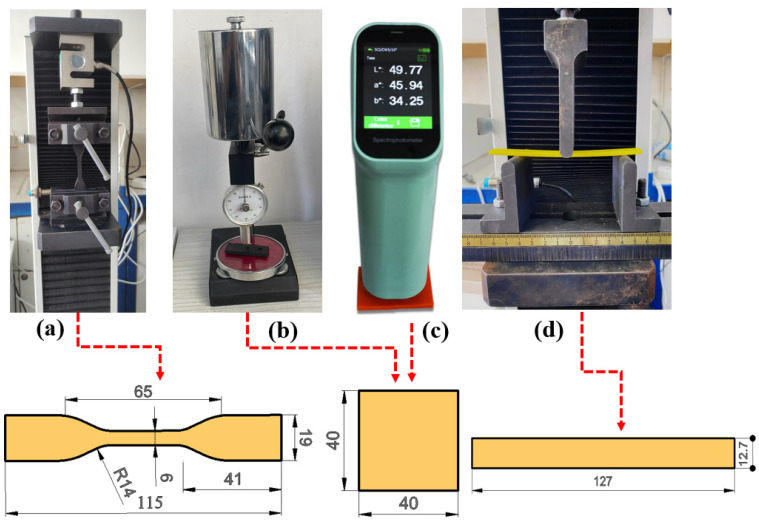
Equipment and sample dimensions used in the study. (**a**) tensile testing, (**b**) hardness, (**c**) color measurement and (**d**) bending testing.

Figure 1 presents the equipment and sample dimensions used to determine the mechanical properties and color change before and after aging. In Figure 1a,d, the WDW-5 model universal tensile testing machine with a 5 kN capacity, used for tensile tests (ASTM D638 [42]) and bending tests (ASTM D790 [43]), along with the sample dimensions, are shown. The tests were conducted at room temperature with a test speed of 2 mm/min and were repeated four times. For hardness measurements, the Loyka brand Shore D hardness tester (Shenzhen Yibai Network Technology Co., Ltd., Guangdong, China) was used, as shown in Figure 1b. Hardness measurements were carried out in accordance with ASTM D2240 standard [44] and repeated ten times, with averages taken. To determine the effect of aging on color, the CHNSpec brand CS 410 model spectrocolorimeter (Chnspec Technology Co., Ltd., Hangzhou, China) was used, as shown in Figure 1c. Measurements were performed three times before and after aging.

## 3. Findings and Discussion

In the study, two different experimental groups were prepared using three different colors (yellow, orange, and red) and three different infill ratios (20%, 60%, and 100%). The first group of samples was considered the reference group, while the second group was subjected to aging. The color, hardness, tensile strength, and bending force of all samples were compared. As a result, the effect of filament colors on mechanical properties before and after aging, as well as the relationship between color and strength, were discussed.

### 3.1. Mechanical Properties Before Aging

#### 3.1.1. Tensile Strength in Unaged Parts

**Figure 2:**
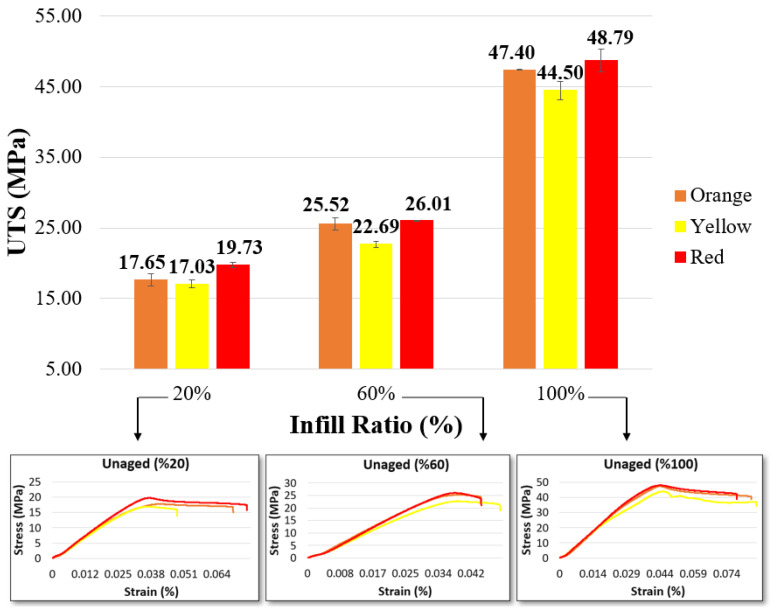
Tensile test values of unaged materials.

In Figure 2, the ultimate tensile strength (UTS) and stress–strain graphs of the unaged samples are presented. The stress–strain diagrams in Figure 2 are drawn from the dataset that best represents the average value. For all infill ratios (20%, 60%, and 100%), the highest UTS values were observed in the red samples, recorded as 19.73 MPa, 26.01 MPa, and 48.79 MPa, respectively. Gao et al. compared the mechanical properties of PLA and ABS parts with a 100% infill ratio and eight different colors. In their study, the best values for tensile, bending, and compressive tests were found for purple-colored PLA, with values of 52.5 MPa, 65.9 MPa, and 62 MPa, respectively. For ABS parts, the highest tensile strength was recorded at 24.9 MPa, and the highest compressive strength at 43.9 MPa for red samples, while the highest flexural strength was 35.9 MPa for green samples [25]. This study demonstrates that the colorants added to filaments for different colors directly impact mechanical properties. Additionally, various color pigments from different manufacturers are used to color filaments in the industry. Gao et al. noted that although the mechanical properties of the filaments were known, it was difficult to interpret the results of the study because the manufacturers kept the chemical contents secret. In another study, filaments with orange and black colors were produced at three different infill ratios (20%, 60%, and 100%) to investigate the effect of color on mechanical properties. The highest tensile strengths were reported for black samples at 53.7 MPa for the 100% infill ratio, 19.26 MPa for orange, and 19.61 MPa for black at the 60% infill ratio, and 16.8 MPa for orange samples at the 20% infill ratio. The critical point noted was that black samples performed better at the 100% infill ratio, the tensile performance of orange and black samples was similar at the 60% infill ratio, and orange showed higher performance at the 20% infill ratio [45]. Frunzaverde et al. investigated the effects of filament color and printing temperature on mechanical properties, surface roughness, and dimensional accuracy. For this purpose, they tested PLA filaments in two different colors (natural and black) at five different printing temperatures (200–240 °C). As a result, the highest UTS for natural PLA was recorded as 50.41 MPa at 230 °C, while for black PLA, the highest UTS was 52.3 MPa at 200 °C [7]. These results indicate that mechanical properties vary in parts printed with different colors at different infill ratios.

In the results of the current study, the colors of the parts with the highest tensile strength were identified as red, orange, and yellow, for all infill ratios. Notably, red and yellow are primary colors, while orange is an intermediate color produced from these two [46]. According to the data, the strength of orange was found to be between that of red and yellow. The percentage elongation values of the red and orange samples were relatively similar but higher compared to the yellow samples (Table 3). In light of this information, 3D printer users should consider that the mechanical strength of parts varies depending on the color when producing their prints.

Table 4 presents the fracture surfaces of unaged samples after the tensile test. When the fracture types of the samples were evaluated based on infill ratios, they were found to be similar. However, in samples with 20% and 60% infill ratios, the fractures were observed to be angular and zigzag-shaped. In contrast, in samples with a 100% infill ratio, the fractures occurred perpendicular to the load direction. In these cases, the fracture type was sharper and more linear. The findings of the fracture profiles of the tensile test samples after testing are consistent with the literature [47,48].

#### 3.1.2. Bending Results Before Aging

**Figure 3:**
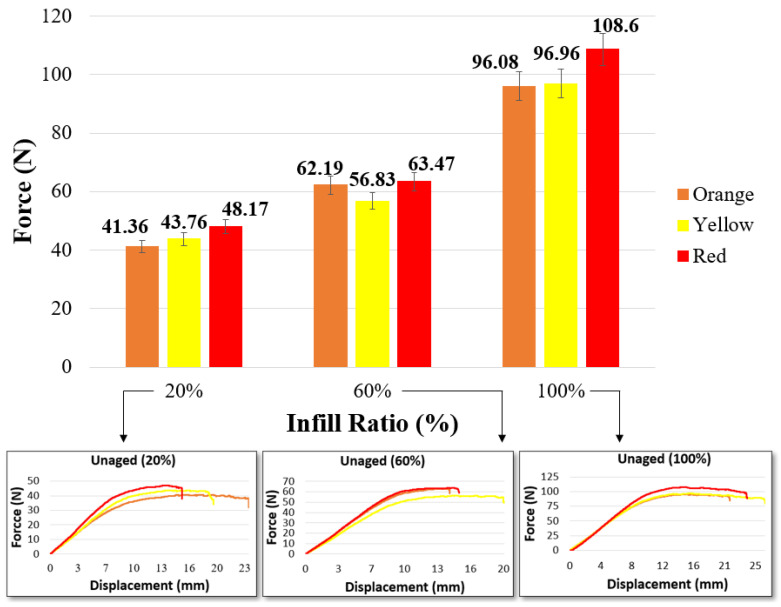
Bending test values of unaged materials.

Figure 3 and Table 5 show the bending graphs of the samples according to their color and infill ratio. In all samples, the bending force increased proportionally with the infill ratio. Similarly, Öteyaka et al. investigated the effects of different infill ratios (0%, 20%, 40%, 60%, and 100%) on the flexural strength of PLA. As a result, they stated that the flexural strength of PLA increased as the infill ratio increased, reaching a maximum of 35.65 N (0%: 12.68 N, 20%: 16.98 N, 40%: 18.13 N, 60%: 25.05 N, 80%: 32.19 N) at a 100% infill ratio [49]. Akhoundi et al. investigated the effect of different infill ratios (20%, 50%, and 100%) and different patterns (concentric, Hilbert curve, rectilinear, and honeycomb filling) on the mechanical properties of PLA. They reported that the flexural strength increased with the infill ratio, and the best flexural strength for all infill ratios was obtained with the concentric pattern [50]. In addition, Gao et al. determined that the bending force varied depending on the color in their study with eight different colored filaments at a 100% infill ratio. The highest bending force was 65.9 MPa in purple, and the lowest bending strength was 52.5 MPa in natural color [25].

In this study, similar to the findings in the literature, at a 20% infill ratio, the bending force ranged between 41.36 N and 48.17 N; at a 60% infill ratio, it ranged between 56.83 N and 63.47 N; and at a 100% infill ratio, it ranged between 96.08 N and 108.6 N. At all infill ratios, the bending force of the red samples was higher than that of the orange and yellow samples. In the red samples, the bending force increased by 16% at a 20% infill ratio, 11.68% at a 60% infill ratio, and 13.03% at a 100% infill ratio. Table 5 presents the maximum bending force values. As the bending force increases, the deformation on the surface begins earlier. The highest displacement was recorded as 21.6 mm in the orange sample at a 20% infill ratio, 18.89 mm in the yellow sample at a 60% infill ratio, and 26.11 mm in the yellow sample at a 100% infill ratio.

### 3.2. Mechanical Properties After Aging

#### 3.2.1. Tensile Strength After Aging

**Figure 4:**
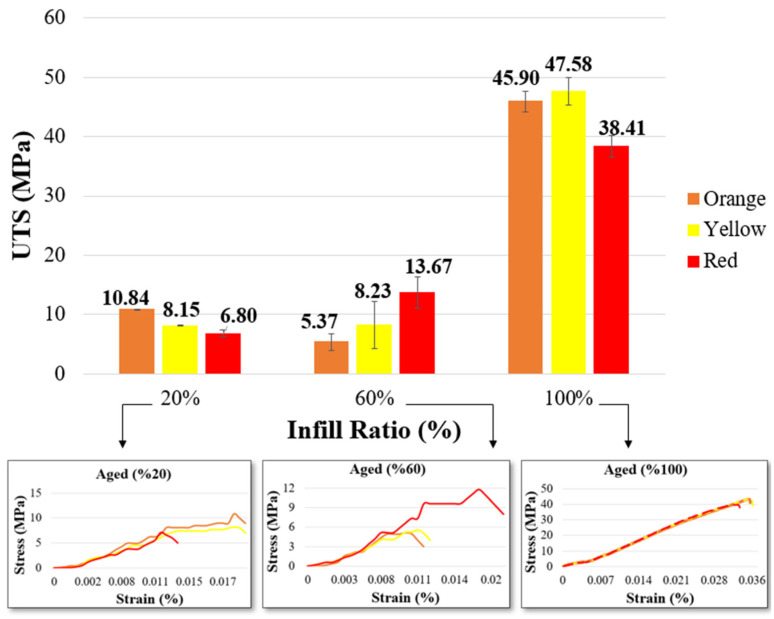
UTS and stress–strain graphs of aged material.

In Figure 4, the UTS and stress–strain graphs of aged material samples after tensile testing are presented. The stress–strain diagrams in Figure 4 are drawn from the dataset that best represents the average value. The highest UTS values obtained, according to 20%, 60%, and 100% infill ratios, were 10.84 MPa, 13.67 MPa, and 47.58 MPa in samples with orange, red, and yellow colors, respectively. In their study, Sedlak et al. examined both the mechanical properties of the same and different filaments from various manufacturers before aging and the material behavior observed after aging. For this, they obtained PLA, PETG, ASA, and ABS filaments from different manufacturers. After conducting an aging experiment under UV light at 400 mm/20 h, they reported yellowing in ABS samples, stickiness on the PLA surface, and that PETG and ASA filaments were unaffected by aging. Under conditions of 400 mm/100 h, color changes in ABS and ASA filaments became more noticeable depending on the brand [13]. Although manufacturers provide the mechanical properties of filaments, the color additives used in production are not disclosed. Mechanical tests on samples of different colors in this study also indicate that aging behavior varies depending on the part’s color, and that colors influence the altered mechanical properties after aging [13]. This demonstrates that the additives used in filament production affect both the mechanical properties and the material behavior after aging.

After aging, the red sample with a 100% infill ratio showed a 21.27% reduction in mechanical properties, the orange sample showed a 3.16% reduction, and the yellow sample displayed a 6.9% increase in mechanical properties. Consequently, among the samples with a 100% infill ratio, the orange samples were less affected by aging compared to the red samples, while the yellow samples showed improvement. In terms of elongation values, the orange sample had higher values at the 20% infill ratio, while the red-pigmented filament showed higher values at 60% and 100% infill ratios (Table 6). The significant impact of aging on red samples is due to the ability of the red color to absorb many wavelengths. Red pigments are primarily associated with blue, which strongly absorbs ultraviolet light [51]. The high-energy photons absorbed cause increased ionization and degradation of the compounds that absorb the energy. In contrast, yellow generally reflects most of the spectrum, reducing the effects of aging.

It is known that materials of the same type but different colors can exhibit varying responses to aging depending on their color [52]. Furthermore, for all polymer materials, an increase in surface temperature accelerates the rate of photo-oxidation. When colored products are exposed to radiation, the surface temperature varies significantly based on the color. The surface temperature of the sample largely dictates the speed of the aging process. The photo-oxidation of plastic due to solar UV radiation makes it more susceptible to degradation [53]. Studies examining the surface temperatures of colored plastic products exposed to sunlight under the same conditions have shown that red-colored samples have higher surface temperatures compared to yellow ones [18]. This is one of the reasons for the observed reduction in strength.

Table 7 shows the fracture surfaces of aged samples. When comparing the fracture types in these samples based on infill ratios and their unaged counterparts, they appear quite similar. Additionally, all samples exhibit significantly more shape distortion compared to unaged samples. In aged samples with 20% and 60% infill ratios, fractures occurred in an angled and zigzag pattern. Fracture lines are particularly more distinct in the yellow and orange samples. For samples with a 100% infill ratio, the fractures are perpendicular to the direction of the applied load, resulting in a straight fracture pattern. The fracture appearances of the samples are consistent with the relevant literature [54].

**Figure 5:**
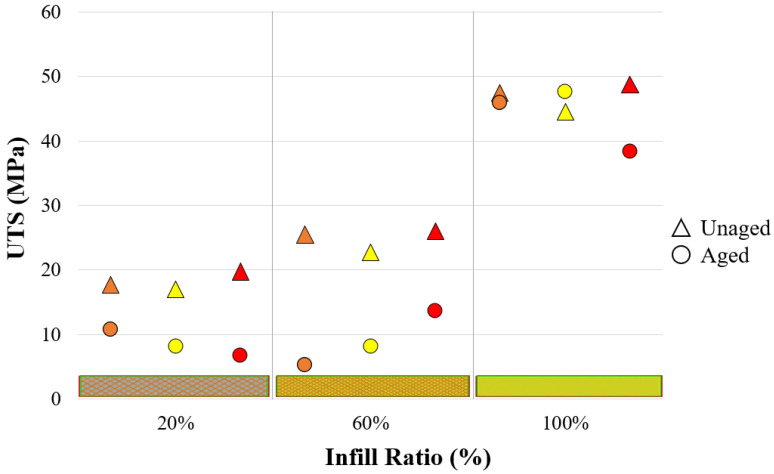
Comparison of tensile strengths of unaged and aged material.

Figure 5 compares the tensile strengths of unaged and aged samples. At a 100% infill ratio, when comparing unaged and aged samples, the tensile strength in the orange samples was very similar (47.40/45.90 MPa), aged yellow samples had higher tensile strength (44.50/47.58 MPa), and aged red samples showed lower tensile strength (48.79/38.41 MPa). In aging studies found in the literature, Bergaliyeva et al. examined the effects of aging based on two different aging methods and time durations. In their experimental study, they applied accelerated thermal aging to PLA at 50 °C for various durations (8, 16, 24, 48, 72, 168, 672, 1344 h) and hydrothermal aging (24, 48, 72, 168, 672, 1344 h). Tensile strength gradually increased up to 24 h of aging compared to the unaged material. All thermally aged samples showed better mechanical properties than the unaged material. In hydrothermally aged samples, the best mechanical properties were achieved at 24 h (33.45 MPa), while the lowest were recorded at 1344 h (20.89 MPa) [8]. Similar to Bergaliyeva et al.’s findings, an increase in tensile strength was observed in yellow samples after aging in the current study. Studies indicate that mechanical properties vary depending on color [25,55]. In this study, mechanical tests also reveal that colors affect the samples’ responses to aging. This can be explained by different colors absorbing different wavelengths (ultraviolet) and exhibiting varying molecular vibrations (temperature) [56]. The colors of the samples in this study absorbed UV light to different degrees during aging, causing each sample to reach a unique temperature based on its color. Consequently, the effects of aging varied from sample to sample.

When evaluated by infill ratios, it was observed that the effect of aging increased significantly in orange and yellow samples at 20% and 60% infill ratios. The reason for this is that at 20% infill ratio, there are more voids, which increases light transmittance. With increased light transmittance, the parts heat up less, reducing the impact of thermal shocks. In red samples, the aging effect was similar across all infill ratios. This is due to the influence of the red pigment, which absorbs different wavelengths on the part’s surface, making thermal shocks more impactful.

Table 8 presents the change in hardness values of the samples after aging. The hardness differences between aged and unaged samples range from 0.48% to 13.5%. The lowest hardness change was observed in the orange sample with a 100% infill ratio, while the highest hardness change was found in the orange sample with a 20% infill ratio.

#### 3.2.2. Bending Results After Aging

**Figure 6:**
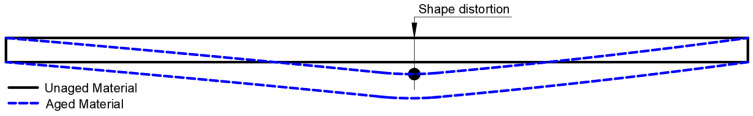
Distortion formation in bending samples after aging.

The distortion observed in all bending samples after aging was analyzed. The bending deformation of the aged and unaged samples is shown in Figure 6. Table 9 provides images of the bending samples after aging. Distortion occurred in all three colors at all infill ratios. Table 10 presents the measurement results from all samples in millimeters. The highest distortion (5.5, 6.75, and 3.5 mm) was measured in red samples across all infill ratios. When the red samples are assessed, the highest distortion was measured as 7 mm in parts with a 60% infill ratio. This may be attributed to the higher light transmittance in samples with a 20% infill ratio due to their structure, while the structural integrity at a 100% infill ratio reduces the aging effect. Orange samples were the least affected by aging.

**Figure 7:**
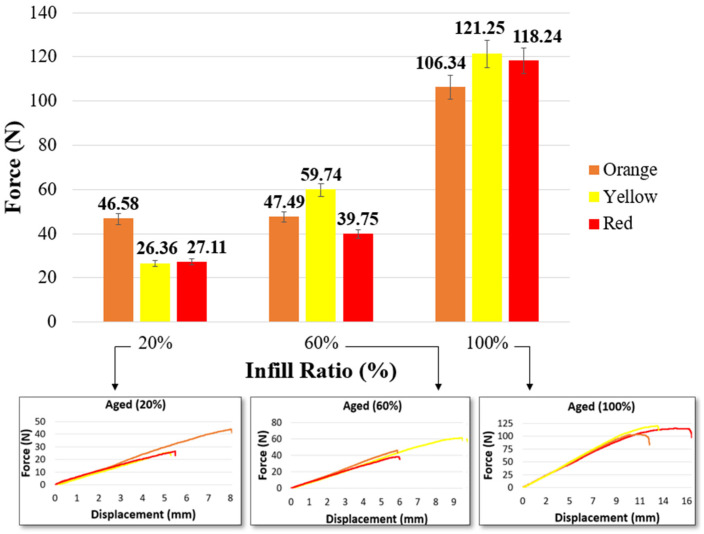
Bending test results of aged materials.

Figure 7 and Table 11 present the bending forces of samples after aging, based on color and infill ratio. At a 20% infill ratio, the bending force increased by 12.6% in orange samples, while it decreased in red and yellow samples. At a 60% infill ratio, the bending force increased by 5.12% in yellow samples, while it decreased in red and orange samples. At a 100% infill ratio, the bending forces increased by 10.37%, 25.05%, and 8.87% in orange, yellow, and red samples, respectively. When the displacements are examined in Table 11, it can be determined that cracks occurred earlier in the aged samples compared to the unaged samples. The latest crack onsets were determined as 9.17 mm in the orange sample at a 20% infill ratio, 10.00 mm in the yellow sample at a 60% infill ratio, and 15.30 mm in the yellow sample at a 100% infill ratio. After aging, bending results at a 60% and especially 100% infill ratio showed better performance than the tensile results.

### 3.3. Color Difference After the Aging of Polymers and the Relationship Between Color and Strength

In the experiments, PLA materials in three different colors (orange, red, and yellow) were used. Color measurements were conducted on all samples (both aged and unaged) in accordance with the ASTM D2244 standard [57].

**Figure 8:**
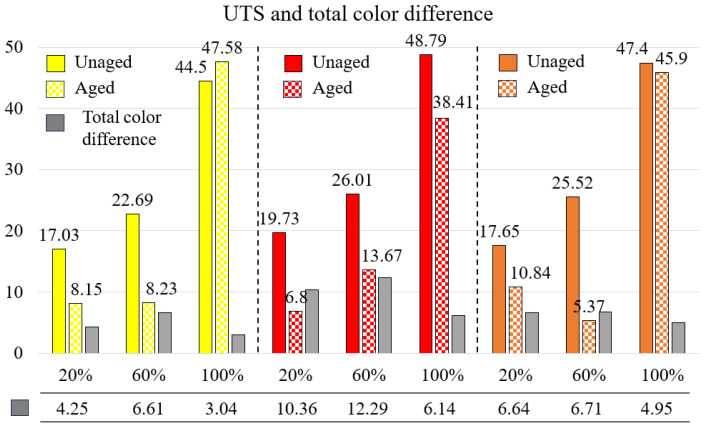
The relationship between UTS and total color difference.

Visual inspections were conducted by comparing the colors of the aged and unaged samples. The CIELAB color space parameters were then determined, and the results were analyzed. The L*, a*, and b* values of each sample were measured, and averages were calculated. The values of the unaged and aged samples, obtained from these measurements that aimed to determine the effect of aging on the total color change (∆E*), along with the average values of their differences, are provided in Table 12. Figure 8 shows the UTS of both aged and unaged samples, as well as the total color difference between them.

In visual inspections, the colors of the aged samples were observed to differ from those of the reference materials. The highest color changes were observed in red specimens with 20% and 60% infill ratios, where all aged red samples showed signs of fading. The lowest color change was recorded in yellow samples with a 100% infill ratio. For all colors, the total color change in samples with 100% infill ratio was lower than in samples with 20% and 60% infill ratios. This is thought to be due to the fact that materials printed with a 100% infill ratio do not have internal voids, helping to preserve their integrity during aging. In contrast, aging had a greater impact on samples with internal gaps, resulting in more significant color changes.

When comparing the total color difference at 100% infill ratios, the total color difference between aged and unaged red samples was the highest among the three colors. The tensile strength of the red samples decreased with aging, suggesting a linear relationship between color difference and strength degradation due to aging. Among the samples with a 100% infill ratio, the total color changes from highest to smallest were as follows: red (6.14), orange (4.95), and yellow (3.04). The tensile strength of the yellow samples increased after aging, with yellow showing the smallest color difference. In the orange samples, the tensile strength remained similar before and after aging. In red samples, the difference in tensile strength between aged and unaged samples increased significantly after aging. Across all infill ratios, the color difference increased as the tensile strength difference between aged and unaged samples grew.

For aged samples with a 100% infill ratio, the bending force was higher than in unaged samples. For unaged samples with 20% and 60% infill ratios, the bending forces were similar to each other. After aging, lighter-colored samples are expected to exhibit less color change. In this study, yellow samples showed the least color difference. Aged yellow samples with a 100% infill ratio had higher tensile strength and bending force than unaged ones. The strength can be positively affected after aging of samples with colors that are assumed to have low color change, because aging causes different deteriorations in polymer materials of different colors.

**Figure 9:**
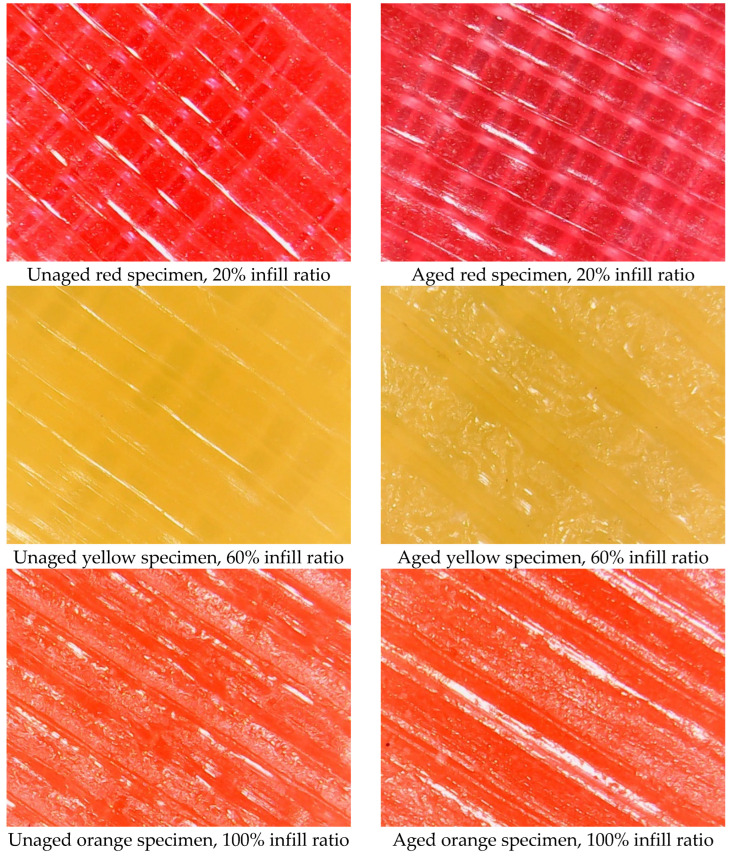
Surface images of selected aged and unaged specimens.

Figure 9 provides images of the surfaces of aged and unaged test samples. Nieto et al. immersed PLA and PETG samples in three different solutions (sea salt, sugar-saturated water, and distilled water) for 70 days. They observed a color change (yellowing) in all samples compared to reference samples. Additionally, they observed transparency in PLA, likely due to a hydrolysis process where dipolar water molecules tend to break the polymer’s carbon–hydrogen bonds [28]. Similarly, in the current study (Table 12), color changes (transparency and whitening) were observed. Transparency was particularly noticeable in red samples with a 20% infill ratio.

## 4. Conclusions

This study investigates the changes in color and mechanical properties of 3D-printed PLA with different colors and infill ratios after aging, as well as the relationship between these properties. The findings obtained from aging PLA in different colors indicate that the functionality (mechanical and physical properties) in long-term applications depends on the optical properties (colors) of the materials.

In all sample colors used in the study, mechanical properties increased as the infill ratio increased. Additionally, the mechanical properties of the samples were found to vary depending on the color. At all infill ratios (20%, 60%, and 100%), the highest UTS (19.73 MPa, 26.01 MPa, and 48.79 MPa) and bending forces (48.17 N, 63.47 N, and 108.6 N) were observed in red samples.

The 3D-printed PLA materials were more affected by aging at lower infill ratios. After aging, the red samples with a 20% infill ratio show a 65.53% reduction, and the orange samples with a 60% infill ratio show a 78.9% reduction. At a 100% infill ratio, yellow samples show a 6.9% increase, while orange and red samples show reductions of 3.16% and 21.27%, respectively. It was determined that at a 100% infill ratio, orange samples were less affected by aging, while red samples were more affected. The increase in tensile strength of the yellow sample after aging can generally be explained by the fact that it reflects most of the light falling on it.

After aging, the bending forces increased by 12.6% in orange samples at a 20% infill ratio, by 5.12% in yellow samples at a 60% infill ratio, and by 10.37%, 25.05%, and 8.87% in orange, yellow, and red samples, respectively, at a 100% infill ratio.

The highest color changes after aging were observed in red samples with 20% and 60% infill ratios, while the lowest color change was calculated in yellow samples with 100% infill ratio. As the infill ratio increased, the total color change between aged and unaged samples decreased. This is because the hollow parts are more affected by aging. It was determined that as the color difference between aged and unaged samples increased, the mechanical properties changed. In aged colored samples, low color difference changes may correlate with higher strength.

During 3D printing, structural changes occur in PLA, and there are some uncertainties regarding material transformations. The additives and colorants that manufacturers include in PLA are not disclosed. Therefore, it is important for researchers studying 3D printer filaments to consider that the sources of commercial materials are unclear [58,59]. Furthermore, manufacturers often list the same properties for a given material type in the technical data sheet without considering color selection [25]. For components at high risk of UV damage, design and material selection should aim to minimize UV degradation. In industrial applications, UV-resistant plastics or secondary treatments such as coatings or protective surface finishes can be employed to ensure UV protection. The findings of this study aim to provide insights into the relationship between color changes and mechanical properties of 3D-printed PLA specimens subjected to accelerated aging under laboratory conditions. In the future, similar studies on non-PLA 3D filament materials could broaden the scope of research in this area. Ultimately, as additive manufacturing materials are exposed to a wide range of operating conditions (e.g., varying weather conditions), further experimental studies are needed to evaluate their long-term performance. Additionally, colored polymer materials may undergo different degradations under various aging conditions. Detailed studies with different colors and aging conditions (including varying durations and other parameters) would help bridge the gap in understanding the relationship between color change and strength.

## Figures and Tables

**Table 1:** Technical properties of filament material [38].

Mechanical Properties	PLA
Density (g/cm^3^)	1.24
Tensile strength (MPa)	60
Tensile elongation (%)	6
Flexural strength (MPa)	83
Rockwell hardness (R-scale)	108

**Table 2:** The 3D printing parameters.

Layer thickness (mm)	0.2
Nozzle temperature (°C)	205
Table temperature (°C)	60
Print speed (mm/sn)	50
Printer nozzle diameter (mm)	0.4

**Table 3:** Tensile strength and % elongation values of unaged materials.

Infill Ratio	20%		60%		100%	
Color	UTS (MPa)	Strain (%)	UTS (MPa)	Strain (%)	UTS (MPa)	Strain (%)
*Orange*	17.65 ± 0.91	6.40	25.52 ± 0.87	4.95	47.40 ± 0.05	9.11
*Yellow*	17.03 ± 0.55	4.81	22.69 ± 0.42	4.84	44.50 ± 1.29	8.10
*Red*	19.73 ± 0.33	7.73	26.01 ± 0.02	4.64	48.79 ± 1.62	8.72

**Table 4:** Fracture surfaces of samples after tensile.

Color	Infill Ratio		
	20%	60%	100%
Orange	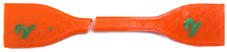	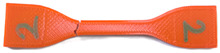	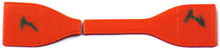
Yellow	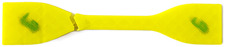	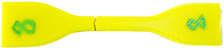	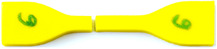
Red	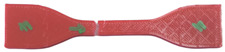	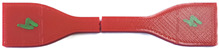	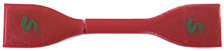

**Table 5:** Force and displacement values of unaged materials.

Infill Ratio	20%		60%		100%	
Color	Force (N)	Disp. (mm)	Force (N)	Disp. (mm)	Force (N)	Disp. (mm)
*Orange*	41.36 ± 1.31	21.60 ± 2.51	62.19 ± 1.36	15.02 ± 1.02	96.08 ± 1.69	20.91 ± 1.08
*Yellow*	43.76 ± 1.07	17.46 ± 1.94	56.83 ± 1.99	18.89 ± 1.01	96.96 ± 2.04	26.11 ± 1.26
*Red*	48.17 ± 1.91	16.40 ± 0.32	63.47 ± 1.51	15.17 ± 0.31	108.60 ± 2.27	22.82 ± 1.41

Disp.: displacement.

**Table 6:** Tensile and elongation values of aged materials.

Infill Ratio	20%		60%		100%	
Color	UTS (MPa)	Strain (%)	UTS (MPa)	Strain (%)	UTS (MPa)	Strain (%)
*Orange*	10.84 ± 0.08	2.01	5.37 ± 1.43	1.17	45.90 ± 3.78	3.39
*Yellow*	8.15 ± 0.10	1.78	8.23 ± 3.99	1.43	47.58 ± 5.67	2.98
*Red*	6.80 ± 0.62	1.51	13.67 ± 2.65	1.98	38.41 ± 1.80	3.90

**Table 7:** Fracture surfaces of samples after aging.

Color	Infill Ratio		
	20%	60%	100%
Orange	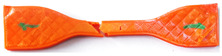	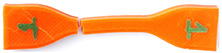	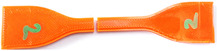
Yellow	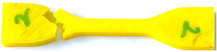	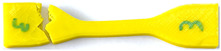	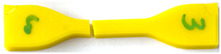
Red	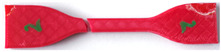	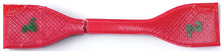	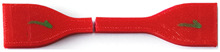

**Table 8:** Hardness change after aging.

Color	Condition	Orange	Difference	Yellow	Difference	Red	Difference
Infill Ratio							
20%	Unaged	48.0 ± 0.8	−13.5%	42.3 ± 1.3	+12.3%	46.1 ± 0.4	−1.3%
	Aged	41.5 ± 3.5		47.5 ± 4.9		45.5 ± 6.5	
60%	Unaged	77.5 ± 1.2	+8.7%	74.0 ± 2.1	+4.8%	78.0 ± 2.1	−11.5%
	Aged	84.3 ± 2.0		77.6 ± 0.9		69.0 ± 1.1	
100%	Unaged	82.5 ± 0.2	−0.48%	84.8 ± 1.0	−1.4%	85.8 ± 0.2	−3.8%
	Aged	82.1 ± 0.2		83.1 ± 0.2		82.5 ± 0.7	

**Table 9:** Images of bending samples after aging.

Color	Infill Ratio		
	20%	60%	100%
Orange	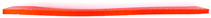	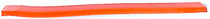	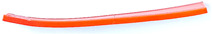
Yellow	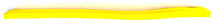	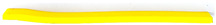	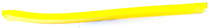
Red	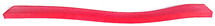	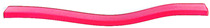	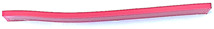

**Table 10:** Maximum distortion distances in bending samples after aging (mm).

	Sample Number	20%	60%	100%
Orange	1	1.5	1.5	1.6
	2	1.0	0.75	1.5
	3	2.0	1.0	1.4
Yellow	1	1.9	1.5	1.5
	2	1.75	1.0	3
	3	1.8	2.0	1
Red	1	3.0	6.75	3.5
	2	5.5	4.5	2.5
	3	4.25	7.0	0.5

**Table 11:** Force and displacement values of aged samples.

Infill Ratio	20%		60%		100%	
Color	Force (N)	Disp. (mm)	Force (N)	Disp. (mm)	Force (N)	Disp. (mm)
*Orange*	46.58 ± 4.00	9.17 ± 1.85	47.49 ± 2.43	6.22 ± 0.96	106.34 ± 4.29	12.34 ± 0.22
*Yellow*	26.36 ± 1.95	5.34 ± 0.40	59.74 ± 3.78	10.00 ± 0.19	121.25 ± 1.68	13.08 ± 1.21
*Red*	27.11 ± 1.17	7.16 ± 1.71	39.75 ± 2.69	5.56 ± 0.94	118.24 ± 4.41	15.30 ± 2.45

**Table 12:** Color values of unaged and aged samples with different colors and infill ratios.

Color		Yellow			Red			Orange		
Infill Ratio		20%	60%	100%	20%	60%	100%	20%	60%	100%
Unaged materials	L_1_*	73.57	77.78	79.48	38.51	39.23	38.54	49.58	49.42	49.84
	a_1_*	−2.57	1.37	3.12	39.77	39.84	38.72	44.30	49.37	47.78
	b_1_*	65.87	73.69	75.35	16.60	16.84	16.55	33.70	38.90	35.54
Aged materials	L_2_*	71.44	76.51	78.49	41.76	43.55	40.55	50.16	50.81	50.58
	a_2_*	−1.74	−1.33	3.18	34,89	37.08	38.61	38.19	44.01	43.70
	b_2_*	62.29	67.81	72.47	8.06	5.71	10.75	31.17	35.10	32.84
ΔE*		**4.25**	**6.61**	**3.04**	**10.36**	**12.29**	**6.14**	**6.64**	**6.71**	**4.95**

## Data Availability

The original contributions presented in this study are included in the article. Further inquiries can be directed to the corresponding author.
